# Individuals Who Believe in the Paranormal Expose Themselves to Biased Information and Develop More Causal Illusions than Nonbelievers in the Laboratory

**DOI:** 10.1371/journal.pone.0131378

**Published:** 2015-07-15

**Authors:** Fernando Blanco, Itxaso Barberia, Helena Matute

**Affiliations:** 1 Labpsico, Departamento de Fundamentos y Métodos de la Psicología, Universidad de Deusto, Bilbao, Spain; 2 The Event Lab, Facultat de Psicologia, Universitat de Barcelona, Barcelona, Spain; 3 Departament de Psicologia Bàsica, Facultat de Psicologia, Universitat de Barcelona, Barcelona, Spain; Universidad de Granada, SPAIN

## Abstract

In the reasoning literature, paranormal beliefs have been proposed to be linked to two related phenomena: a biased perception of causality and a biased information-sampling strategy (believers tend to test fewer hypotheses and prefer confirmatory information). In parallel, recent contingency learning studies showed that, when two unrelated events coincide frequently, individuals interpret this ambiguous pattern as evidence of a causal relationship. Moreover, the latter studies indicate that sampling more cause-present cases than cause-absent cases strengthens the illusion. If paranormal believers actually exhibit a biased exposure to the available information, they should also show this bias in the contingency learning task: they would in fact expose themselves to more cause-present cases than cause-absent trials. Thus, by combining the two traditions, we predicted that believers in the paranormal would be more vulnerable to developing causal illusions in the laboratory than nonbelievers because there is a bias in the information they experience. In this study, we found that paranormal beliefs (measured using a questionnaire) correlated with causal illusions (assessed by using contingency judgments). As expected, this correlation was mediated entirely by the believers' tendency to expose themselves to more cause-present cases. The association between paranormal beliefs, biased exposure to information, and causal illusions was only observed for ambiguous materials (i.e., the noncontingent condition). In contrast, the participants' ability to detect causal relationships which did exist (i.e., the contingent condition) was unaffected by their susceptibility to believe in paranormal phenomena.

## Introduction

Despite the availability of scientific knowledge and efforts to develop a knowledge-based society, paranormal beliefs remain common. For example, a 2005 poll indicated that 37% of Americans believed in "haunted houses" and 27% of UK citizens thought that it is possible to communicate mentally with dead people [1]. In 2010, two in five Europeans claimed to be superstitious according to the European Commission [2]. It remains unknown why many individuals maintain supernatural beliefs while others are skeptical.

Different related terms (e.g., paranormal, supernatural, magical, etc.) have been used to refer to the same type of beliefs [3]. We will use the term "paranormal belief" as a general label. Deficits in intelligence [4] and in critical/statistical thinking [5] have been proposed to underlie individual differences in paranormal beliefs. However, these claims have been criticized on methodological grounds [6], and empirical tests generated mixed or controversial results [7,8]. For instance, receiving courses on research methods or statistics, both of which imply critical reasoning, does not affect by itself the endorsement of paranormal beliefs unless the course is accompanied by specific guidance and tutorials to grant some degree of transfer [9,10].

On the other hand, paranormal beliefs correlate strongly with reported personal paranormal experiences [11]. This finding suggests that a factor related to the way believers behave or interpret reality, such as a type of generalized bias, leads to a belief in paranormal claims. Some research lines assume that paranormal beliefs are the result of biased thinking, sometimes related to personality traits. For example, Eckblad and Chapman [12] proposed that one of the traits accompanying high schizotypy is the proneness to interpret personal experiences as paranormal ("magical ideation"). More recent research [13] has gone further to conceptualize this magical ideation bias, characteristic of the mentioned personality trait, as a tendency to make a "false-positive" error when testing hypotheses, just like a scientist who commits a Type-I error. Related works also drew a simile with a biased response criterion [14,15].

In a similar vein to the latter authors, it has been proposed that a contingency learning bias underlies many, widely spread, irrational beliefs such as those involved in pseudomedicine or pseudoscience [16]. Instead of focusing on individual differences in personality traits or psychopathology, this contingency learning approach is based on a laboratory model that reveals the bias in the general population. This contingency learning bias is known as "the illusion of causality", or "causal illusion".

Causal illusions arise when people systematically interpret the ambiguity in random patterns of stimuli as evidence of a causal relationship. For example, no objective evidence indicates that wearing a lucky charm causes a desired outcome (e.g., winning a match), but individuals may be inclined to interpret ambiguous information (e.g., cases in which one wears the amulet and plays well) as compelling evidence favoring the cause-effect link. This bias allows fast and efficient detection of causality based on co-occurrences, at the cost of developing illusory beliefs occasionally. While paranormal beliefs are typically measured using questionnaires, illusions of causality are studied using contingency learning experiments in which participants see a series of occurrences of a potential cause and an outcome. Sometimes the trials are predefined by the researchers, but normally, participants decide in which trials they want to introduce the potential cause. In both variants of the task, the contingency between the potential cause and the outcome is fixed by the experimenter to a null value (i.e., the participant should conclude that no causal link exists), but the frequency of cause-outcome coincidences is also manipulated to induce the illusory perception of a causal link [16,17].

We can draw a parallel between paranormal beliefs and causal illusions. In fact, paranormal beliefs have been previously described as the result of a biased causal inference: Brugger and Graves [13] conceive paranormal beliefs as a form of reasoning based on invalid assumptions about causality (see also [12,15,18]). Moreover, evidence indicates that the illusion of control, a type of illusion of causality in which the illusory cause is the participant's behavior, correlates reliably with paranormal beliefs [19]. We suggest that surveys and questionnaires that measure paranormal beliefs might reveal the results of illusions of causality that participants developed in the past via the same mechanisms that are studied in contingency learning experiments. Consequently, the finding that some individuals exhibit more irrational beliefs than others, as measured using questionnaires or other methods, might indicate not only that they developed illusions in the past, but also that they have a stronger tendency to develop illusions of causality. If this is true, they should show a stronger vulnerability to laboratory-induced illusions of causality, as compared to nonbelievers. A similar strategy for studying other proposed origins of paranormal beliefs has been used by many researchers [18–20].

We can further refine our prediction by postulating a candidate mechanism for the vulnerability to develop causal illusions. The evidence to support our following proposal comes from studies on paranormal beliefs and on contingency learning. First, Brugger and Graves [13] found that participants with high scores on a magical ideation scale tested fewer hypotheses to solve an experimental problem and relied on confirmatory evidence more often than participants with low scores. That is, they showed a prominent hypothesis-testing bias, sampling confirmatory information more often than disconfirmatory information. Then, returning to the contingency learning literature, we point out that the bias in information sampling that Brugger and Graves [13] reported is very similar to a very particular pattern of behavior that is often reported in the contingency learning literature when the participant is given the opportunity to decide in which trials to introduce the potential cause. This pattern of behavior implies observing more cause-present information than cause-absent information. In fact, we have documented a preference in participants to expose themselves to the potential cause very often [21]. That is, when asked to evaluate the relationship between a fictitious medicine and a frequent, but unrelated, recovery from a fictitious disease, our participants exposed themselves predominantly to cause-present events (i.e., cases in which the medicine was administered), rather than sampling equal numbers of cause-present and cause-absent events. This biased behavior is in some way parallel to the one documented by Brugger and Graves [13] in paranormal believers. In addition, the consequence of the biased behavior is similar to that of the positive testing strategy [22], an strategy that involves performing tests that would yield a positive answer (i.e., recovery) if the initial hypothesis (i.e., that the medicine is effective) were correct, although in our experiments the biased behavior might be occurring because of other reasons. For example, the participants could just be trying to obtain the outcome (i.e., recovery from the disease), which would not imply necessarily a hypothesis-testing strategy. Consequently, throughout this paper we will use the term "biased exposure to information" to refer more neutrally to the finding that people expose themselves more to cause-present cases than to cause-absent cases. After all, regardless of the participants' motivation to behave in this way, the critical consequence is their exposure to a biased sample of information extracted from all the potential cases that can be observed.

Being exposed to biased information in this particular way has consequences in what refers to causal judgments. We recently demonstrated that the degree of exposure to the potential cause of an outcome influences illusions of causality [17]. In our studies, when the actual contingency between the potential cause and the outcome was zero (i.e., when no causal relationship should be derived from the available information), those participants showing a greater exposure to cause-present cases developed a stronger illusion of causality. This, we suggest, is mainly due to the following mechanisms: even when the actual contingency remains close to zero, people tend (a) to expose themselves more often to cause-present trials, and (b) to put more weight on those situations in which both the potential cause and the outcome co-occur [23,24]. Therefore, a heavier weight of cause-outcome co-occurrences combined with the already mentioned bias in the exposure to information (exposure to more cause-present cases) would lead to a stronger overestimation of the contingency. Then, if believers in the paranormal actually experience more cause-present cases in the contingency learning task (as we propose), the tendency of believers to develop stronger causal illusions would be partly mediated by the bias in the information to which they expose themselves.

To sum up, we suggest that individual differences in the way people behave and expose themselves to available information (like those observed by Barberia et al [21] and Brugger and Graves [13] in their respective paradigms and literatures) leads to different proneness to develop new illusions of causality in the laboratory (in fact, this relationship has been replicated several times in what concerns contingency learning [17,25]). Then, those people with a marked tendency to produce causal illusions would be probably more inclined to attribute the occurrence of certain random events of their lives to spurious causes not only in the laboratory, but also in contexts related to the paranormal (e.g., reading their horoscope in a newspaper). These attributions would eventually crystallize as paranormal beliefs that can be measured in a questionnaire. Unfortunately, in a typical laboratory setting, we are unable to test this latter step directly because, according to the view we have just exposed, paranormal beliefs result from a long previous history of experiences that is unique to each individual. This renders our proposal that paranormal beliefs originate as causal illusions speculative. However, we can readily measure currently held paranormal beliefs and examine how new illusions of causality appear in a contingency learning task in the laboratory, to test whether paranormal believers are more likely to develop illusions of causality. This has been the typical approach when studying related hypotheses about the relationship between biases in causal reasoning and paranormal beliefs [18–20].

With exploratory aim, we also included three additional questionnaires to assess locus of control, desire for control, and attitude towards science. Both the locus of control scale [26] and the desire for control scale [27] have been reported to correlate with paranormal beliefs (with believers showing more internal locus of control [28–30] and stronger desire for control [31]). In addition, an intuitive prediction is that both questionnaires would correlate with causal illusion, because displaying an illusion in our contingency learning task amounts to attributing control to produce outcomes to one's own decisions. Finally, we included the attitude toward science scale [32] because we presumed that people with negative attitude towards science would be more prone to have paranormal beliefs and also to fall prey to causal illusions.

## Method

### Ethics statement

The ethical review board of the University of Deusto examined and approved the procedure used in this experiment, as part of a larger research project (Ref: ETK-44/12-13). All participants signed a written informed consent form before the session.

### Participants and Apparatus

Sixty-four psychology students from the University of Deusto (52 female), with a mean age of 18.69 years (*SD* = 1.45; range from 18 to 26) received extra credit for participating in this study. The experiment was conducted in two separate computer classrooms simultaneously.

### Procedure

The session included two activities: a computerized contingency learning task and a set of four paper-and-pencil questionnaires. Approximately half of the participants in each room completed the contingency task prior to the questionnaires. The order was reversed for the remaining participants. In addition, the order of the questionnaires was counterbalanced.

#### Contingency task

Similar to the conventional contingency learning task [33], participants were asked to play the role of medical doctors and judge the ability of the fictitious medicine Batatrim to cure the fictitious illness Lindsay Syndrome. The participants viewed a series of 40 medical records (one per trial) describing patients suffering from the illness on a computer screen. In each trial, the participants decided whether or not to administer the medicine to the current patient. After making their decision, the participants received feedback indicating whether the patient was cured. We recorded the proportion of trials in which participants chose to administer the medicine, P(Cause), as a measure of the tendency to bias the information they sample during the task. After observing all 40 patients, the participants judged the effectiveness of the medicine using a scale ranging from 0 (labeled as "ineffective") to 50 (labeled "moderately effective") to 100 (labeled "perfectly effective"). The effectiveness judgment is actually an expression of the perceived strength of the causal relation between the medicine (i.e., the potential cause) and the healings (i.e., the outcome) [16,34,35]. The participants then studied the ability of a second medicine (Dugetil), to cure Hamkaoman Syndrome using the same procedure.

One of the medicines was completely ineffective, (i.e., cures were noncontingent on the participants' decision to use the medicine: P(Outcome|Cause) = P(Outcome|¬Cause) = 0.75. In contrast, the other medicine was highly effective (i.e., the contingency between medicine usage and cures was positive: P(Outcome|Cause) = 0.75 and P(Outcome|¬Cause) = 0.125, making the contingency 0.625. Given that judgments should be close to zero in the noncontingent condition, if judgments were significantly higher than zero, that would indicate an illusion of causality. Moreover, because judgments of contingency are typically accurate in nonzero contingencies [36,33], the positive contingency condition served as an additional control to distinguish between actual illusions of causality and indiscriminate high judgments in the noncontingent condition. The order of presentation of the noncontingent and contingent conditions was counterbalanced between subjects.

#### Questionnaires

Participants completed four questionnaires: the Spanish version of the Revised Paranormal Belief Scale, R-PBS [37,38]; the Spanish version of the Locus of Control scale [26,39]; Spanish version of the Desirability for Control scale [27,40]; and the Attitude toward Science scale [32]. All scales except the R-PBS were included with exploratory purposes.

The R-PBS is widely used to assess paranormal beliefs and consists of 30 statements (e.g., "Witches do exist.") covering a range of paranormal beliefs. Eight subscales were identified in the Spanish version [38]: religion, psychic phenomena, witchcraft, traditional superstitions, spiritualism, monsters, precognition, and extraterrestrial visitors. The statements are rated by the participant on a scale from 1 (i.e., "Complete disagreement") to 7 (i.e., "Complete agreement").

The Locus of Control scale [26,39] contains 23 items, each of them consisting of two statements (labeled A and B), one representing an internal locus of control and another representing an external locus of control. For instance, Item 1 comprises the statements "A. Most sad things that happen to people are due to bad luck" and "B. Bad things that happen to people are due to their own mistakes". The participant must choose one of the statements for each item, the one he or she feels more identified with. By counting the number of statements that represent internal and external control attitudes, one can compute an overall locus of control score for the participant.

The Desire for Control scale [27,40] is a set of statements that must be assigned one value from 1 ("Complete disagreement") to 5 ("Complete agreement"). The statements reference a range of situations over which the person may like to exert control (e.g., "I prefer a job in which I have control over what I do and when I do it"). The higher the score in this questionnaire, the stronger the desire for control of the participant.

Finally, the Attitude toward Science scale [32] is a collection of statements such as "Thanks to science, we have a better world to live in", that must be rated on a 5-point scale from "Total agreement" to "Total disagreement". The scale was included because we expected it to be negatively correlated with paranormal beliefs and illusions of causality.

## Results

(The dataset on which the following analyses were conducted is freely available as supporting materials S1 Dataset).

We first report the mean P(Cause) for both contingency problems. In the noncontingent problem, the participants decided to use the medicine frequently (*M* = 0.72, *SD* = 0.26), but less often than in the contingent problem (*M* = 0.82, *SD* = 0.16), *F*(1, 63) = 9.56, *p* = 0.003, $\eta_{p}^{2}$ = 0.13. This difference is discussed later.

Likewise, the mean effectiveness judgments measured in the noncontingent problem (*M* = 54.42, *SD* = 25.56) were significantly lower than those measured in the contingent problem (*M* = 70.34, *SD* = 15.08), *F*(1, 63) = 25.45, *p*<0.001, $\eta_{p}^{2}$ = 0.29. These results indicate that the participants discriminated between the two contingencies and realized that the causal relationship was stronger in the contingent problem. In addition, they also show that the noncontingent problem was perceived as contingent (mean judgments were higher than zero, *t*(63) = 17.04, *p*<0.001), indicating that a causal illusion developed.

Table 1 shows the correlation matrix between the scores obtained on the R-PBS and its subscales and the variables assessed in the contingency task. The R-PBS score and several subscales correlated positively with the judgments and the P(Cause) values obtained in the noncontingent problem. Importantly, these scales did not correlate with any variable obtained in the contingent problem (minimum p-value = 0.125) in which no illusion was expected.

**Table 1:** Observed correlations between the variables assessed in the two contingency learning problems (columns) and the paranormal belief scores (rows).

	Noncontingent problem		Contingent problem	
	Judgment	P(Cause)	Judgment	P(Cause)
PBS-Total	*0.284	**0.388	-0.064	0.115
	(0.023)	(0.002)	(0.618)	(0.365)
PBS-Religion	0.222	*0.316	-0.115	0.194
	(0.078)	(0.011)	(0.365)	(0.125)
PBS-Psi	0.106	*0.273	-0.106	-0.095
	(0.403)	(0.029)	(0.406)	(0.453)
PBS-Witchcraft	*0.298	*0.305	0.054	0.043
	(0.017)	(0.014)	(0.670)	(0.737)
PBS-Superstition	0.105	0.162	-0.096	0.153
	(0.409)	(0.202)	(0.450)	(0.227)
PBS-Spiritualism	0.245	**0.395	-0.088	0.143
	(0.051)	(0.001)	(0.490)	(0.258)
PBS-Monsters	0.112	0.129	-0.023	0.154
	(0.378)	(0.308)	(0.858)	(0.225)
PBS-Precognition	*0.262	*0.294	0.043	0.087
	(0.036)	(0.018)	(0.736)	(0.494)
PBS-ETs	0.187	0.162	0.020	0.002
	(0.139)	(0.200)	(0.873)	(0.987)

The top number in each cell corresponds to the Pearson's coefficient. Exact p-values are provided between brackets.

* *p* < 0.05;

** *p* < 0.01.

We used the method proposed by Judd, Kenny and McClelland [41] to test the interaction between R-PBS scores and problem (contingent vs. noncontingent) on the judgments, to find that the difference between the two slopes was significant, β = 0.33, *t*(62) = 2.71, *p* = 0.009. This interaction was then examined within each problem: the effect of the R-PBS on the judgments was present in the noncontingent problem, β = 0.28, *t*(62) = 2.34, *p* = 0.02, but not in the contingent problem, β = -0.06, *t*(62) = 0.50, *p* = 0.62. The same analyses were conducted on P(Cause), showing similar results: a significant interaction between R-PBS and problem, β = 0.32, *t*(62) = 2.69, *p* = 0.009, with significant effect of R-PBS on P(Cause) in the noncontingent problem, β = 0.39, *t*(62) = 3.31, *p* = 0.002 and nonsignificant effect in the contingent problem, β = 0.11, *t*(62) = 0.91, *p* = 0.36. All these analyses align with the conclusions drawn from Table 1, which suggested that paranormal beliefs were related to P(Cause) and judgments only when the programmed contingency was zero.

**Fig 1:**
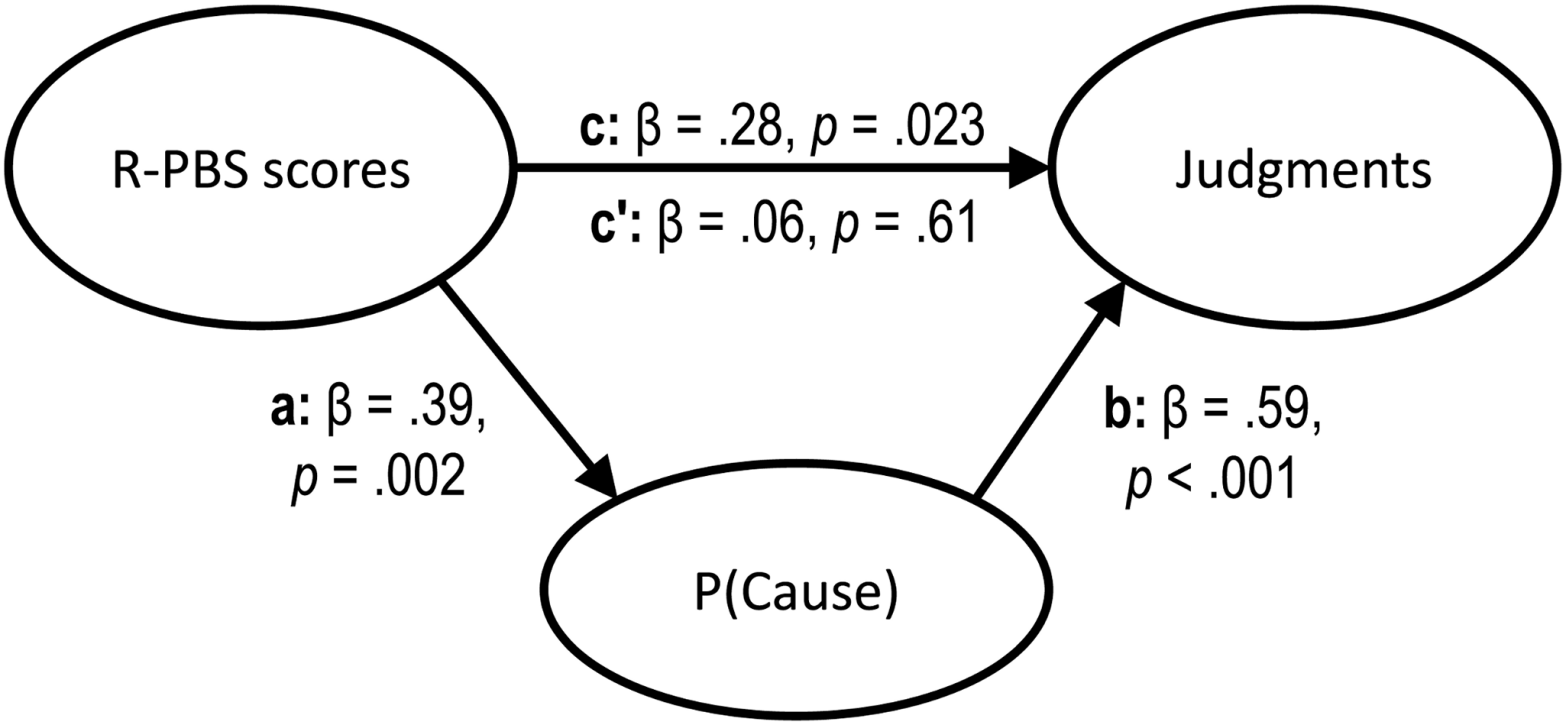
Mediational structure tested in the noncontingent condition. The total effect of the paranormal belief (R-PBS) scores on judgments (depicted as pathway c) was partitioned into one indirect effect via P(Cause) (pathways a and b), and one direct effect (pathway c'), which is the result of discounting the indirect effect. The results of this study suggest that prior paranormal beliefs increased the tendency to develop new causal illusions via the mediation of a biased exposure to cause-present information.

**Fig 2:**
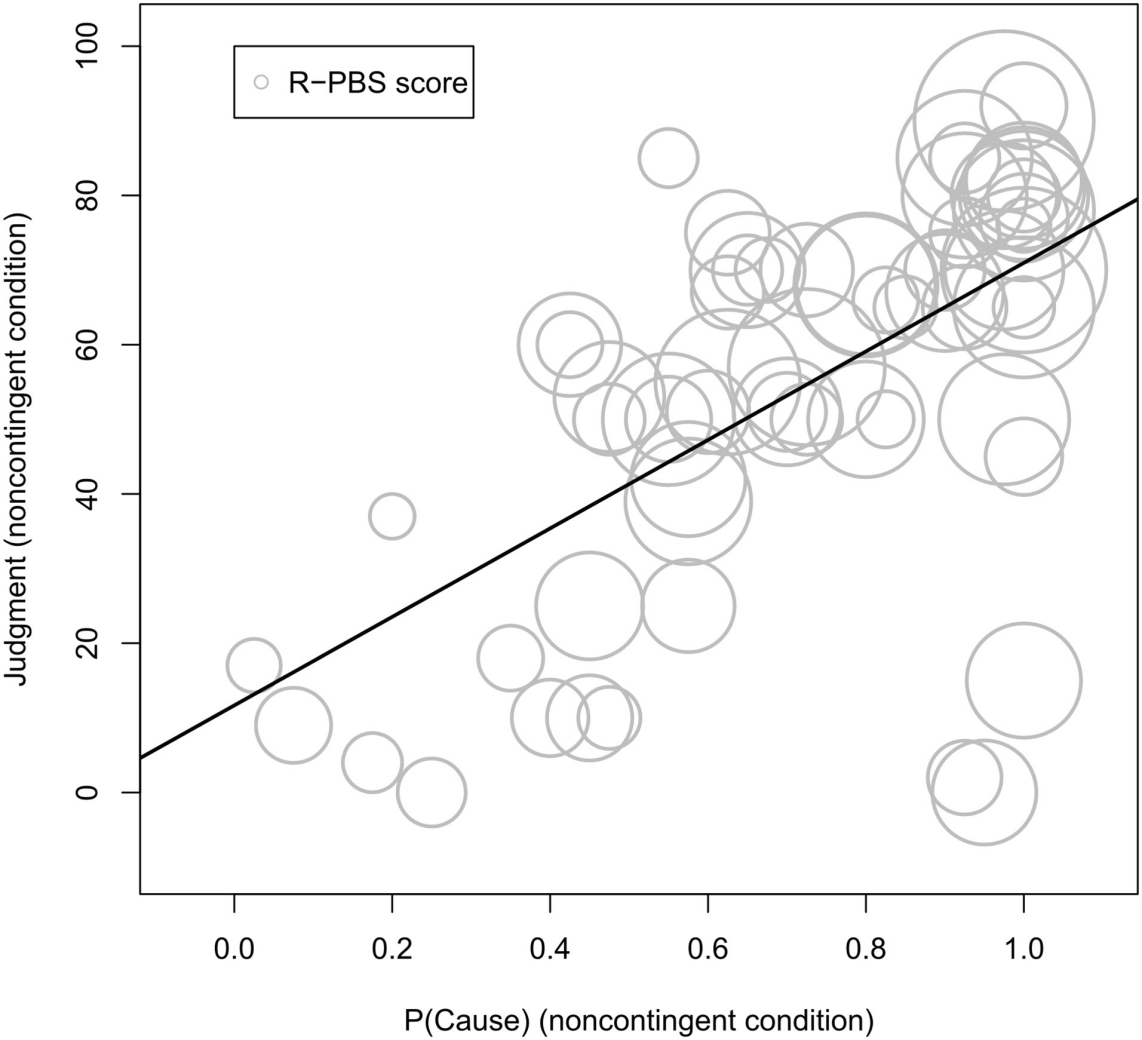
Bubble chart showing the positive relationship between P(Cause) (horizontal axis), Judgments (vertical axis) and R-PBS scores (circle area) in the noncontingent condition. A regression line was fitted to the P(Cause)-Judgments relationship (see main text).

Thus, participants with higher paranormal belief scores developed stronger causal illusions in the noncontingent problem. Next, we examined the potential role of P(Cause) as a mediator of this effect (see the mediational structure presented in Fig 1), so that people with more paranormal beliefs would introduce the cause more frequently and this biased behavior would in turn strengthen the illusion of causality. To this aim, we used the procedure described by Baron and Kenny [42]. At a descriptive level, we show in Fig 2 that higher levels of P(Cause) imply both higher judgments and higher scores in the R-PBS, which is consistent with the mediation hypothesis. The indirect pathway between paranormal beliefs and judgments via P(Cause) (Fig 1, paths a and b) was assessed in two steps. First, we found that paranormal beliefs were a positive predictor of P(Cause), β = 0.39, *t*(63) = 3.31, *p* = 0.002 (i.e., the more beliefs the participants held, the more likely they were to use the ineffective medicine often during the contingency learning task) (Fig 1, pathway a). Second, P(Cause) was a reliable predictor of the causal illusion, even when we controlled for the effect of paranormal belief scores, β = 0.59, *t*(62) = 5.33, *p*<0.001 (Fig 1, pathway b). Thus, the indirect effect of paranormal beliefs on judgments via P(Cause) was significant (see Fig 2). When this indirect effect was partialled out, the direct effect of paranormal beliefs on judgments disappeared, β = 0.06, *t*(63) = 0.52, *p* = 0.61 (Fig 1, pathway c’), suggesting that the effect of paranormal beliefs on judgments was mediated entirely by P(Cause).

**Fig 3:**
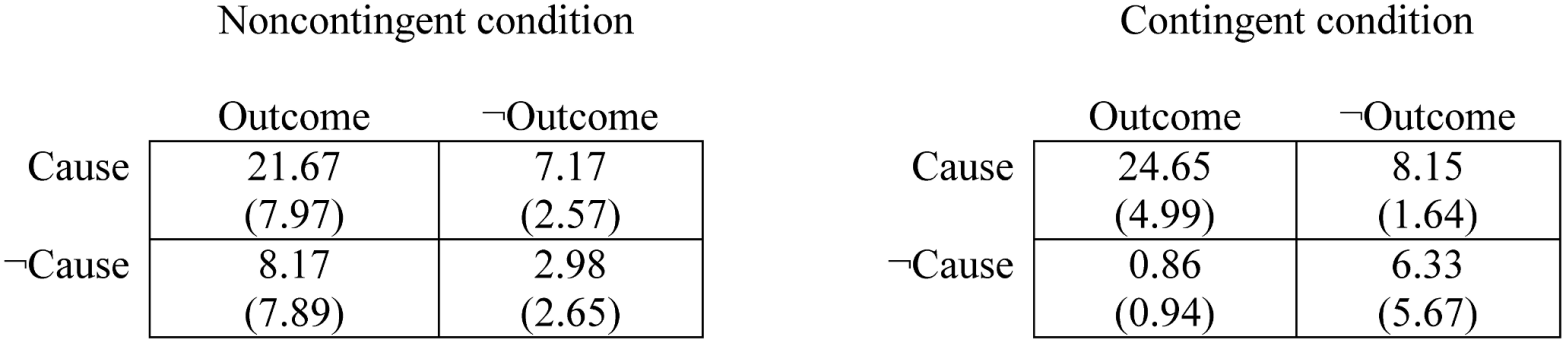
Mean frequencies of each trial type to which the participants exposed themselves in each condition. Standard deviations are provided between brackets.

Previous studies [17,43] have shown that, in this type of active tasks in which the participant is free to decide how often and when to introduce the potential cause (i.e., the medicine), there is some variability in the actual contingency that participants expose themselves to, and sometimes it could differ from the programmed contingency. However, the mean value of the actual contingency is usually close to the programmed value. For completeness, we include the two contingency tables with the mean frequencies of each type of trial the participants were exposed to in each problem (see Fig 3). From these frequencies the actual contingency can be computed using the ΔP rule. These actual contingencies were close to the programmed ones in the noncontingent problem (*M* = 0.05, *SD* = 0.23) and in the contingent problem (*M* = 0.64, *SD* = 0.14).

In addition, we report the observed correlations between the variables assessed in the contingency task and the remaining exploratory questionnaires in Table 2. A positive attitude towards science correlated negatively with judgments in both contingency problems, which suggests that participants with positive attitude tended to be more skeptical even in the condition in which the contingency was positive. Both the Desire for Control and the Locus of Control scales failed to correlate with any variable in the contingency learning task.

**Table 2:** Observed correlations between the variables assessed in the two contingency learning problems (columns) and the three exploratory questionnaires (rows).

	Noncontingent problem		Contingent problem	
	Judgment	P(Cause)	Judgment	P(Cause)
Desire for control	0.178	0.007	0.173	-0.011
	(0.158)	(0.958)	(0.172)	(0.934)
Attitude toward science	*-0.278	-0.043	**-0.468	-0.037
	(0.026)	(0.733)	(<0.001)	(0.773)
Locus of Control (the more positive, the more internal)	0.182	0.000	0.174	-0.014
	(0.150)	(0.998)	(0.169)	(0.911)

The top number in each cell corresponds to the Pearson's coefficient. Exact p-values are provided between brackets.

* *p* < 0.05;

** *p* < 0.01.

Finally, the correlations between the scores obtained in these three exploratory questionnaires were not significant except for the following ones: Desire for Control was negatively related to positive attitude towards science (*r* = -0.39, *p* = 0.002) and to Locus of Control (the more internal the locus, the higher the desire for control; *r* = 0.99, *p*<0.001).

## Discussion

We previously proposed [16] that the prevalence of paranormal beliefs in society is associated to a general bias toward perceiving a causal link where no evidence exists (i.e., an illusion of causality). Consistent with this hypothesis, the results of the current experiment revealed a significant correlation between the previous paranormal beliefs of the participants and the magnitude of the illusion of causality that they developed in the laboratory, in a task that used fictitious medicines and illnesses and was, therefore, unrelated to their beliefs. Importantly, paranormal beliefs correlated with judgments only in the noncontingent problem, suggesting that the ability to detect a causal relation when evidence exists (i.e., in the contingent problem) was unaffected by paranormal beliefs.

We also measured the proportion of trials in which the participants decided to use the medicine, P(Cause). Believers were more likely to use it. Thus, they acted in a way that exposed them to cause-present trials more predominantly than did nonbelievers. This parallels previous reports from a different research line and using a different experimental procedure, in which paranormal believers sampled more confirmatory information [13]. However, with the present study we cannot know the reasons why believers tended to expose themselves to high levels of P(Cause), it could be due to the same information sampling strategy reported by Brugger and Graves [13], or simply a stronger tendency to persist in obtaining the outcome, for example.

The relationship between paranormal beliefs and judgments in the noncontingent task was mediated entirely by this biased exposure to the available information. Thus, we propose that believers developed stronger causal illusions due to their tendency to bias their behavior to experience more cause-present cases. The idea that individual differences in personality traits affect the development of causal illusions is not novel. For example, mildly depressed participants are less likely to report illusions in a contingency learning paradigm [44,45]. This depressive realism effect appears to be mediated by the same information-exposure bias described in this study (i.e., depressed people are less likely to act, and therefore they experience less cause-present cases than do nondepressed people [45]).

Admittedly, the mediational structure tested in the Results section is only one among the many that can be hypothesized. We assumed that the participants' paranormal beliefs were a preexisting condition instead of being the outcome of the study, whereas the P(Cause) and the judgments were produced in a definite moment in the laboratory. The performance measured in the contingency learning task was in fact affected by individual differences in paranormal beliefs: believers tended to sample more cause-present trials and showed greater illusions during the experiment. Therefore, we were not testing how paranormal beliefs originated in our participants. Instead, we tested the hypothesis (suggested in the literature: [13,16,19]) that paranormal believers would be more prone to the illusion of causality, and that the mechanism mediating this proneness would be a bias in the way they behave and, therefore, a bias in the information they are exposed to, so that more cause-present than cause-absent cases are experienced. However, although we do not need to assume that all paranormal beliefs originate as causal illusions, it seems sensible to propose that those individuals with greater vulnerability to causal illusions developed this type of illusions in the past, in other situations that, unlike our laboratory settings, are related to paranormal phenomena (e.g., when reading their horoscope in a newspaper). Then, this would reveal as a correlation between paranormal belief and causal illusion in our experiment. In fact, previous works resting on the hypothesis that paranormal beliefs are produced by certain traits (e.g., deficits in the ability to make probabilistic judgments) used designs similar to ours, in which paranormal beliefs are measured and then an experimental task is conducted [18].

It is worth noting that the medical scenario in the contingency task that we used was unrelated to the paranormal domain. Believers still biased the information they observed in the noncontingent problem, so that they were exposed to more cause-present than cause-absent cases, and developed a causal illusion. This finding suggests that the observed bias is a general bias that is not restricted to the paranormal domain. It seems that believers in the paranormal are biased in a way that makes them more likely to develop illusions in general (i.e., not only those related to paranormal beliefs). This finding may have important societal implications, as the illusion of causality is proposed to underlie other problems, including the spread of pseudoscience, vulnerability to advertisement, social stereotypes, and intolerance [46].

Another point that needs clarification is the high proportion of cause-present cases, P(Cause), that was observed in the contingent problem as compared to the noncontingent problem, irrespective of the paranormal beliefs. According to previous studies [21], it is actually very common to find higher probability of cause-present trials when the programmed contingency is positive. In addition, we do not necessarily interpret this finding as the result of the same type of bias that we reported in the noncontingent scenario. First, because the desired outcome (i.e., recovery from the disease) was contingent on the use of the medicine in this problem, this behavior of using the medicine was reinforced more frequently than its counterpart (i.e., not using the medicine), and thus it became prevalent. Second, the contingent problem is not as ambiguous as the noncontingent problem: once the contingency is learned by the participant (i.e., they realize that the medicine actually works), the most probable behavior is to use the medicine frequently to produce the positive outcome (i.e., healing the patients) as often as possible. These two conditions are not met in the noncontingent problem, in which the desired outcome was not produced by the participants' behavior, and still they preferred to use the medicine with high probability, and even more often when they held paranormal beliefs.

As commented in the Results section, despite having some variability in the probability with which the participants decided to use the medicine, the actual values of the contingencies experienced by them were on average very similar to the programmed values (Fig 3). This is a common finding in the literature of contingency learning [17,43]. Given that our participants were actually exposed to a contingency close to zero in the noncontingent problem, how could they exhibit causal illusions? The way in which most theories explain causal illusions when actual contingency is zero implies the assignment of different weights to each trial type. It is often reported that participants give more importance to those trial types in which the potential cause and the outcome coincide [23,24], and this so-called differential cell weighting could account for the illusion of causality [24,47]. Interestingly, weighting differently each type of trial is a rational strategy in many causal inference situations, but the specific rank of trial weights depends on further factors [48,49]. In any case, judgments given in contingent situations (either positive or negative) tend to be close to their actual value, as in our contingent problem, or at least tend to show the correct tendency between different contingency levels. It is mostly under certain conditions of noncontingency (e.g., high probability of outcome-present trials, and high probability of cause-present trials) that participants produce large overestimations, or causal illusions, like those we reported here. Note that, because the tendency to weight the trial types differently is a basic phenomenon, the causal illusion appears in the general population (e.g., Internet users, college students, etc. [50]) just as optical illusions, as we advanced in the Introduction. In addition, it becomes more prominent in those people with tendency to expose themselves to a disproportionate number of cause-present trials, as is the case of nondepressed participants [45], people instructed to obtain as many outcomes as they can [25], and, according to the current study, paranormal believers.

Although both the tendency to believe in actually nonexistent causal links and to bias the information-exposure behavior would seem problematic traits for any organism, they are in fact adaptive in most circumstances, particularly in those resembling our ancestral way of life: after all they seem to have been favored by natural selection during our evolutionary history. In what concerns the bias to attribute random co-occurrence of events to cause-effect relationships (illusion of causality), some researchers have pointed out the beneficial role of this kind of illusions in the adherence of behaviors [51]. For instance, early farming cultures were able neither to produce nor to understand the conditions for the occurrence of important events such as rain. However, the development of superstitions revolving around these events probably helped our ancestors to not give up and persist trying and cultivating their crops. Another reason why causal illusions are adaptive sometimes is that they frequently represent the least-costly mistake [25,52]. Taking an inoffensive branch for a snake would lead to unnecessarily flee and waste energy, whereas making the opposite mistake might result in death. In these ancestral environments, attributing random patterns to actual causation is often harmless, compared to the consequences of missing a real causal link. Arguably, this advantage of the biased causal reasoning is substantially reduced, or even reversed, in other scenarios. In today's society, important decisions, such as determining which treatment we undergo to cure a disease, should be based on scientific evidence rather than on our natural tendency to identify accidental coincidences between taking the treatment and symptom remission as evidence for the effectiveness of the treatment. Thus, the same biases that help us make quick decisions today and that were valuable tools to survive in the past are sometimes harmful, given that they can lead to dangerous practices (e.g., taking a useless medicine instead of an effective one). Some researchers favor this viewpoint according to which superstitions and other beliefs are natural, unavoidable by-products of an otherwise adaptive learning strategy, which in fact keeps being adaptive in many situations but leads to dangerous consequences in many other ones [53]. The challenge is then to find the optimum level of flexibility to reach a balance between quick learning and caution in judging causal relationships.

There are two methodological points that are worth being commented. In the contingency learning literature, learning is assessed by means of judgments collected at the end of the training session, just as we did in our study. Available evidence indicates that the wording of the question can affect substantially the judgment given by the participant [54,55]. In our case, we chose to formulate the question in terms of effectiveness (i.e., how effective the medicine was to heal the patients), instead of in terms of causality (i.e., how likely the medicine was the cause of the healings), because this wording seems easier to understand, more intuitive for participants, and more ecological, while retaining basically the same meaning. In fact, judgments of effectiveness are more common in the illusion of control literature, but they are also present in causal learning experiments [34,35], even if they are referred to as "causal judgments". At least one study compared the two types of question, effectiveness and causality, and found that they were not significantly different from each other, showing the same basic effect of causal illusion [16]. Thus, not only the effectiveness question is conceptually similar to the causal question, but it yields similar results empirically.

The second concern about the judgment we used to assess the illusion refers to the potential confound between the causal strength perceived by the participants and their confidence in their judgments. This possibility is sometimes called "the conflation hypothesis" [56], and to some extent it seems an unavoidable problem: even in experiments specifically tailored to address this issue, still some participants appear to contaminate their causal judgment by expressing their confidence [56]. Consequently, the potential conflation affects to most of the literature in the causal learning field. In our current study, if participants really confounded effectiveness and confidence in their judgments, that would be problematic only if confidence varied systematically with paranormal beliefs. In addition, when we asked the judgments, we labeled not only the ends of the scale, but also the midpoint, 50, as "moderately effective", which should help participants make the right interpretation. A possible follow-up for our study would explore different judgment scales, such as bidirectional scales, that are also affected by the potential conflation but have a different meaning for their midpoint.

As reported above, in addition to the R-PBS, other questionnaires were included in the study to complement the main variables of interest. First, the locus of control scale [26] was expected to correlate with the judgments in the noncontingent problem, such that the more internal the locus, the stronger the illusion. We found no evidence of correlation between the locus of control and any variable assessed during the contingency learning task. In addition, previous studies [29,30] reported correlations between superstitious beliefs as assessed by the R-PBS and locus of control, which we failed to replicate. Second, the desire for control scale [27] was included because it measures a construct similar to the locus of control. In fact, both correlated in our study (the more internal the locus, the higher the desire of control), but again this questionnaire failed to correlate with P(Cause) and judgments, and also with the paranormal beliefs (thus, we failed to replicate this finding that has been reported in the literature [31]).

The reasons for including the questionnaire of attitude towards science was that, according to our framework, many pseudoscientific beliefs are associated to causal illusions [16]; therefore, positive attitude towards science would correlate with more accurate perception of causality. Actually, participants with a positive attitude towards science judged the fictional medicine less effective in both contingency problems. This result could be caused by these participants’ generalized skepticism, even when the available evidence supports a causal relationship. Thus, on the basis of this datum, we suggest that holding a positive attitude towards science does not improve discrimination between contingent and noncontingent problems, but rather promotes overall conservative judgments.

## Conclusions

In conclusion, believers in the paranormal showed a behavioral bias that produced high exposure to cause-present information and development of new causal illusions of arbitrary content in the laboratory. The results presented here suggest that beliefs in the paranormal are accompanied by a biased exposure to available information, which might fuel causal illusions.

## Supporting Information

S1 Dataset Dataset containing the data used in the study. (XLS)
